# Developmental outcomes of HIV-exposed infants in a low-income South African context

**DOI:** 10.4314/ahs.v20i4.25

**Published:** 2020-12

**Authors:** Carmen Cornelia de Beer, Esedra Krüger, Jeannie van der Linde, Renata Eccles, Marien Alet Graham

**Affiliations:** 1 Department of Speech-Language Pathology and Audiology, University of Pretoria, Pretoria, South Africa; 2 Department of Science, Mathematics and Technology Education, University of Pretoria, Pretoria, South Africa

**Keywords:** Developmental outcomes, HIV-exposed infants, low-income context

## Abstract

**Background:**

Effective HIV transmission prevention strategies have led to a growing population of vulnerable HIV- and antiretroviral-exposed infants in sub-Saharan Africa, however uncertainty exists regarding their development.

**Objective:**

To determine the developmental outcomes of HIV-exposed (HE) infants in a low-income South African context, when compared to HIV-unexposed (HU) counterparts.

**Methods:**

In this prospective cross-sectional, group comparison study, the development of 41 HE and 40 HU infants (mean age=8.4 months, SD=2.1 months) from a low-income context was assessed. Caregivers were interviewed using the Vineland Adaptive Behavior Scales, Third Edition (Vineland-3) to evaluate infants' development.

**Results:**

Most HE participants had age-appropriate overall development (90.2%;n=37). Some HE participants, however, presented with delays in domains of communication (9.8%;n=4), daily living skills (2.4%;n=1), socialisation (19.5%;n=8), and motor development (7.3%;n=3). HU participants also demonstrated some domain-specific delays, thus delays were present in both groups. No statistically significant between-group differences regarding development were found.

**Conclusion:**

Findings were reassuring and suggested that HE and HU participants had similar development. Developmental differences may, however, only emerge with age, therefore large-scale longitudinal research is recommended. It is suggested that the entire sample was vulnerable, highlighting the importance of developmental surveillance in low-income contexts, irrespective of HIV and antiretroviral exposure status.

## Introduction

Approximately 37.9 million people are living with Human Immunodeficiency Virus (HIV) worldwide, of which 20.3% reside in South Africa, a lower-middle-income country in sub-Saharan Africa^1^. More than 87% of South African pregnant women living with HIV access antiretroviral treatment (ART) to prevent mother-to-child transmission of HIV^1^. In sub-Saharan Africa, prevention of mother-to-child transmission programs and ART during and after pregnancy have led to a decrease in infant HIV infection rates and a growing population of vulnerable HIV-exposed (HE) infants, who are exposed to HIV and ART, but not necessarily infected with the virus^2^.

Prevention strategies, such as ART, improve maternal health and prevent infant HIV infection^1^, but there are conflicting findings regarding the effect of antiretroviral (ARV) exposure on infant development^3^. ARVs that cross the blood-brain barrier are a concern for early brain development^4^, and it has been reported that ARV exposure could be associated with preterm birth and mitochondrial dysfunction, resulting in neurological and developmental problems^5^. It is conversely suggested, however, that ARVs are safe to use during pregnancy and the perinatal period^3^ and that ante- and postpartum combination ARV exposure has no adverse effects on infant and child development^4^,^6^. Therefore, the impact of prolonged ARV exposure on the development of HE infants is not yet well established^6^.

The impact of HIV exposure on HE infants' development is also not yet clear. It is recognised that HE infants have better motor, cognitive, and language outcomes than HIV-infected infants^7^, but there is a dearth of literature regarding the developmental outcomes of HE infants below 12 months when compared to their HIV-unexposed (HU) counterparts. The majority of available research focuses on the developmental outcomes of HE children older than 12 months of age^2^,^4^,^6^,^8^, possibly because developmental outcomes and delays are not easily recognised in infants and young children, although early identification leads to timeous intervention^9^.

There are also inconsistent findings on the effect of HIV exposure on early development^2^,^4^,^6^,^8^,^10^,^11^. It has been suggested that HE children may have differences in cognitive, motor, and language development, when compared to HU peers^2^,^10^,^11^. In contrast, other studies report that HE and HU children have no significant differences regarding motor development, cognition, and language^4^,^6^,^8^. Consequently, there is still uncertainty about the developmental outcomes of HE infants during late infancy.

Apart from the possible biomedical impact of HIV and ARV exposure on infant development, the social environment in which HE infants grow up, may increase risks for developmental delay^7^. HIV-infected parents typically experience morbidity, reduced productivity, and increased medical expenses, which place families at risk for poverty and lower socio-economic status, in turn affecting infant development^12^. Maternal morbidity may also impair mother-child attachment and limit the provision of care and stimulation to infants, which are essential for optimal child development^13^.

Biomedical and environmental factors place HE infants at risk for developmental delays^12^. Inadequate knowledge regarding their development may hamper health professionals in identifying vulnerable HE infants timeously and implementing preventative early intervention services, before developmental delays are established. Early intervention can provide infants, families and communities with developmental, educational and economic benefits^14^. The aim of this study was to determine the developmental outcomes of infants with HIV and ARV exposure in a low-income South African context, when compared to developmental outcomes of unexposed counterparts.

## Methods

### Setting

Data were collected at a primary healthcare clinic in an urban settlement in South Africa. It is considered a low-income context, as a monthly household income of less than ZAR1600 (US$107) is received by 40.1% of the population, and most residents reside in poverty with a high rate of unemployment^15^. Thirty-nine per cent of homes are informal dwellings with many unplanned homes being illegally constructed and only 35.9% of homes have piped water inside the dwelling^15^.

### Participants

Purposive sampling was used to recruit 81 infants, aged six to 12 months. Infants were included if caregivers were older than 18 years and were able to answer interview questions in English. Infants with pre- and postnatal HIV and ARV exposure were included in the research group (RG), therefore their mothers were HIV-positive and used ARVs during pregnancy. Infants were included in the control group (CG) if they had exposure to neither HIV nor ARVs. Infants with congenital disorders and confirmed HIV-positive statuses were excluded.

### Procedures

This study received approval from the institutional review board and Gauteng Department of Health (783/2018; GP_201812_019). Data were collected prospectively for two months in this cross-sectional, group comparison study. Caregivers provided consent for themselves and their infants to participate. Developmental assessments were conducted by one speech-language pathologist in a private space during routine clinic visits. Subsequent to assessments, all caregivers received feedback and age-specific brochures from the “Learn the Signs. Act Early.” Program, to increase awareness and monitoring of developmental milestones^16^. Necessary referrals were made if concerns regarding development were identified.

### Measures

#### Demographic characteristics

Caregivers were briefly interviewed to obtain demographic and background information. In addition, the infants' medical records were consulted for relevant developmental, medical, and HIV-related information.

### Infant developmental outcomes

The Vineland Adaptive Behavior Scales, Third Edition (Vineland-3) Comprehensive Interview Form^17^ was administered with caregivers. The Vineland-3 assesses the development of adaptive behaviour, which is described as daily functional skills required for personal and social sufficiency^17^,^18^. It relates to the activity and participation aspects of the International Classification of Functioning, Disability and Health framework^19^,^20^. This measure evaluates developmental domains (and subdomains), namely communication (receptive and expressive language), daily living skills (personal skills), socialisation (interpersonal relationships and play skills), and motor skills (gross and fine motor skills)^17^. The Vineland-3 is a standardised, norm-referenced tool that has high internal consistency, good to excellent test-retest and inter-rater reliability, as well as validity in terms of internal structure and content^17^,^18^. The Vineland-3 was developed and normed in the United States^17^, although its previous versions have been used effectively in lower-middle-income countries^21^,^22^, and HE children in South Africa^23^. This tool is commonly used in the diagnosis of a developmental delay^17^.

### Data analysis

The Vineland-3 test items are scored by means of a rating scale, including the scores 0 (never), 1 (sometimes), and 2 (usually); or in some cases 0 (no) and 2 (yes). These scores reflect the frequency to which participants perform certain behaviours without assistance or prompting^18^. Raw scores are calculated and converted to standard scores for domains and scale scores for subdomains. Three of the four domain standard scores, namely communication, daily living skills, and socialisation domain standard scores are used to compute an overall test score, the Adaptive Behaviour Composite. The Adaptive Behaviour Composite and domain standard scores have a mean of 100 and a standard deviation (SD) of 15, whereas the subdomain scale scores have a mean of 15 and a SD of three^17^. Scores of one SD or more below the normative means are interpreted as a developmental delay^17^. Therefore, Adaptive Behaviour Composite and domain standard scores of ≤85 and subdomain scale scores of ≤12 were interpreted as delayed.

Data were analysed using the Statistical Package for the Social Sciences version 25. Descriptive and inferential statistics were used to determine statistically significant differences between the RG and CG. In order to test for normality, the Kolmogorov-Smirnov or the Shapiro-Wilk test statistics may be used. These two tests are the same in that they are both testing for normality, however, the Shapiro-Wilk test is known to have more power in detecting differences from normality^24^. Since the majority of the p-values for the Shapiro-Wilk test, for the different variables under consideration, were less than 0.05, the data is not normally distributed and, accordingly, non-parametric tests were used. Differences in participant characteristics and Vineland-3 results were determined using the Mann-Whitney U and Fisher's Exact tests. Spearman correlations were used to determine associations. All statistical tests were two-sided and p-values of less than 0.05 were considered significant. The hypotheses for the study were as follows:

**Hypothesis 1:**

Ho: There is no statistically significant difference in the demographic characteristics of the participants between the RG and CG.

Ha: There is a statistically significant difference in the demographic characteristics of the participants between the RG and CG.

**Hypothesis 2:**

Ho: There is no statistically significant difference in the amount of delays of the participants between the RG and CG.

Ha: There is a statistically significant difference in the amount of delays of the participants between the RG and CG.

**Hypothesis 3:**

Ho: There is no statistically significant correlation between maternal age and infant development.

Ha: There is a statistically significant correlation between maternal age and infant development.

For Hypothesis 1 and Hypothesis 2, if the p-value is less than 0.05, then the null hypothesis is rejected and there is a statistically significant difference between the RG and the CG. On the other hand, if the p-value is greater than 0.05, then the null hypothesis is not rejected and there is not a statistically significant difference between the RG and the CG. For Hypothesis 3, if the p-value is less than 0.05, then the null hypothesis is rejected and there is a statistically significant correlation between maternal age and infant development. On the other hand, if the p-value is greater than 0.05, then the null hypothesis is not rejected and there is not a significant correlation between maternal age and infant development.

## Results

A sample of 81 participants (mean age = 8.4 months, SD = 2.1 months) was recruited and divided into a RG of 41 HE participants and a CG of 40 HU participants. The software program G*Power version 3.1.9.4 was used to compute the minimum sample size requirement and the achieved power. The minimum sample size required to obtain a power of at least 0.95 is equal to 69. In this study, a sample size of 81 produced an achieved power of 0.977. Home language distribution was Northern Sotho (43.2%), Tsonga (11.1%), Setswana (9.9%), IsiZulu (8.6%), Shona (6.2%), Ndebele (4.9%), Venda (3.7%), Xhosa (3.7%), Southern Sotho (3.7%), SiSwati (2.5%), and Chichewa (2.5%). All caregivers reported English as an additional language and were therefore able to answer interview questions in English.

Inferential statistics were run to test for statistically significant differences between the RG and the CG and the p-values are presented in Table 1. If the p-value is less than 0.05, then the null hypothesis (Hypothesis 1) is rejected and there is a statistically significant difference between the RG and the CG in terms of the demographic variables. On the other hand, if the p-value is greater than 0.05, then the null hypothesis is not rejected and there is not a statistically significant difference between the RG and the CG. There were no statistically significant differences between the RG and CG in terms of age (p=0.622), gender (p=0.176), birth weight (p=0.242), gestational age (p=0.258), and feeding type (p=0.107) [Table 1]. There were also no significant differences between the groups for maternal education level (p=0.872) or substance abuse during pregnancy (p=0.359; p=0.675). The mean age of the RG mothers was significantly higher than that of the CG mothers (p=0.026).

**Table 1:** Demographic characteristics of participants (n=81)

Characteristics		Research Group: HE participants (n=41)	Control Group: HU participants (n=40)	*p*-value^a^
**Infant age (months), mean (SD)**		8.3 (2.1)	8.5 (2.0)	0.622
**Infant gender**	Male, n (%)	13 (31.7%)	19 (47.5%)	0.176
	Female, n (%)	28 (68.3%)	21 (52.5%)	
**Birth weight (grams), mean (SD)**		2962.6 (432.5)	3060.1 (468.6)	0.242
Gestational age*	Full term (≥38 weeks), n (%)	39 (95.1%)	34 (87.2%)	0.258
	Preterm (≤37 weeks), n (%)	2 (4.9%)	5 (12.8%)	
**Infant feeding** **during first six** **months of life**	Exclusive breastfeeding, n (%)	26 (63.4%)	25 (62.5%)	0.107
	Exclusive formula feeding, n (%)	6 (14.6%)	1 (2.5%)	
	Breast- and formula feeding, n (%)	9 (22.0%)	14 (35.0%)	
**Maternal age (years), mean (SD)**		31.2 (5.0)	28.9 (6.0)	0.026**
**Maternal** **education level**	Less than Grade 8, n (%)	1 (2.4%)	1 (2.5%)	0.872
	Grade 9–10, n (%)	6 (14.6%)	5 (12.5%)	
	Grade 11–12, n (%)	28 (68.3%)	25 (62.5%)	
	Tertiary education, n (%)	6 (14.6%)	9 (22.5%)	
**Maternal** **substance abuse** **during pregnancy**	Smoking, n (%)	4 (9.8%)	1 (2.5%)	0.359
	Alcohol or drugs, n (%)	2 (4.9%)	3 (7.5%)	0.675

a *p*-values of the Mann-Whitney U test for the continuous variables and the *p*-values of the Fisher's Exact test for the frequencies (i.e. the counts)

* Missing value in control group, due to non-disclosure of information (n=1)

** Indicates a statistically significant difference between groups (*p*<0.05)

HE: Human Immunodeficiency Virus (HIV)-exposed; HU: HIV-unexposed; SD: standard deviation

All participants (n=81) were accompanied by their primary caregivers, of which 77 (95.1%) were participants' mothers, one (1.2%) was a father, and three (3.7%) were participants' family members such as grandmothers, who were legal guardians. In the RG, 24 (58.5%) mothers initiated lifelong ART before pregnancy and 17 (41.5%) initiated lifelong ART during pregnancy. Thirty-nine (95.1%) RG mothers were using ARVs at the time of data collection, while two (4.9%) RG mothers stopped using ARVs after pregnancy.

The majority of participants from the RG (90.2%, n=37) presented with age-appropriate development, based on the Adaptive Behaviour Composite overall test scores. The Adaptive Behaviour Composite scores of four (9.8%) RG participants and seven (17.5%) CG participants were delayed. It is important to note that the Adaptive Behaviour Composite score is computed using the communication, daily living skills, and socialisation domain standard scores, but not the motor domain standard score. Motor delays were identified in three (7.3%) RG and two (5%) CG participants. Inferential statistics were run to test for statistically significant differences between the RG and the CG and the p-values are presented in Table 2. If the p-value is less than 0.05, then the null hypothesis (Hypothesis 2) is rejected and there is a statistically significant difference between the RG and the CG in terms of the amount of delays. On the other hand, if the p-value is greater than 0.05, then the null hypothesis is not rejected and there is not a statistically significant difference between the RG and the CG. No significant differences regarding the amount of delays were found between the groups (Table 2), since all the p-values are greater than 0.05. In the total sample (n=81), the most delays occurred within the socialisation domain (18.5%; n=15) and its relating interpersonal relationships subdomain (25.9%; n=21). Conversely, the least delays occurred within the motor domain (6.2%, n=5) and its relating gross motor subdomain (7.4%, n=6).

**Table 2:** Comparison of domain- and subdomain-specific delays (n=81)

Domains and subdomains	Amount of delays, n (%)			*p*-value^a^
	Total sample (n=81)	Research Group: HE participants (n=41)	Control Group: HU participants (n=40)	
**Communication**	11 (13.6%)	4 (9.8%)	7 (17.5%)	0.387
**Receptive language**	14 (17.3%)	4 (9.8%)	10 (25.0%)	0.080
**Expressive language**	11 (13.6%)	7 (17.1%)	4 (10.0%)	0.519
**Daily living skills**	6 (7.4%)	1 (2.4%)	5 (12.5%)	0.210
**Personal**	9 (11.1%)	4 (9.8%)	5 (12.5%)	0.775
**Socialisation**	15 (18.5%)	8 (19.5%)	7 (17.5%)	1.000
**Interpersonal relationships**	21 (25.9%)	12 (29.3%)	9 (22.5%)	0.614
**Play**	8 (9.9%)	4 (9.8%)	4 (10.0%)	0.318
**Adaptive Behaviour Composite overall** **test score**	11 (13.6%)	4 (9.8%)	7 (17.5%)	0.670
**Motor**	5 (6.2%)	3 (7.3%)	2 (5.0%)	1.000
**Gross motor**	6 (7.4%)	5 (12.2%)	1 (2.5%)	0.138
**Fine motor**	15 (18.5%)	10 (24.4%)	5 (12.5%)	0.205

a Fisher's Exact test applied

HE: Human Immunodeficiency Virus (HIV)-exposed; HU: HIV-unexposed

Since the maternal age of the RG and CG differed significantly (p=0.026), correlations were run separately for the two groups, to determine whether associations between maternal age and infant development were present. If the p-value is less than 0.05, then the null hypothesis (Hypothesis 3) is rejected and there is a statistically significant correlation. On the other hand, if the p-value is greater than 0.05, then the null hypothesis is not rejected and there is not a statistically significant correlation. For the RG, there were no significant correlations between maternal age and the communication (p=0.474), daily living skills (p=0.536), socialisation (p=0.088), and motor (p=0.881) domain scores; the Adaptive Behaviour Composite score (p=0.126); or any subdomain scores. Interestingly, the maternal age of the CG correlated significantly with the communication (p=0.007) and socialisation (p=0.020) domain scores; the Adaptive Behaviour Composite score (p=0.017); as well as the gross motor subdomain score (p=0.022). All correlations were positive, therefore as maternal age increased, the developmental scores improved. These associations were, however, not enough to result in between-group differences for developmental outcome comparisons.

## Discussion

No statistically significant between-group differences imply that the RG had similar developmental outcomes to that of the CG. The current study concurs with recent research that reported no developmental differences between HE and HU infants and children^4^,^6^,^8^, contrasting to other studies that have found differences regarding their development^2^,^10^,^11^. This finding is reassuring, considering the growing population of HE infants in sub-Saharan Africa^2^, increased ART provision to pregnant women, and prolonged ARV exposure for HE infants^4^. Resilience and the ability to overcome the adverse effects associated with HIV may be attributed to maternal coping strategies, positive parenting, and a good mother-child relationship^23^. The findings are, however, based on a small, young sample and may not be generalised to other communities. Large-scale longitudinal studies are warranted, as it is proposed that developmental differences may be subtle in early life and may only emerge in later childhood^4^,^8^.

The overall prevalence of delays in the total sample (13.6%, n=11) is lower than reported cognitive and socioemotional delays in lower-middle-income countries (35.8%)^25^, but higher than estimates for developmental delays in the United Stated (4.55%)^26^. Delays that occurred in both the RG and CG may suggest that the entire sample was a vulnerable, at-risk group of infants. A possible explanation for this finding is that infants and children from lower-middle-income countries, like the included population, face multiple risks that can potentially hinder development^27^. These risks may include poverty, poor health, malnutrition, violence, alcohol and other substance abuse, as well as insufficient learning and stimulation opportunities^28^. The study did not aim to specifically investigate environmental and socioeconomic factors that may be associated with developmental outcomes, therefore further research is necessary.

The finding that most delays were identified in the socialisation domain, should be interpreted with caution, as the Vineland-3 was not normed and standardised for the South African population^17^. The tool might not be sensitive to possible cultural differences, as socialisation and interactions vary across cultures^29^. Cultural adaptations of standardised assessment instruments, such as the Vineland-3, should thus be considered in future research.

The least delays were identified in the motor domain and its relating gross motor subdomain. Children from low-income South African contexts have been shown to have high gross motor proficiency, perhaps due to high levels of physical activity and outdoor unstructured play^30^.

Not all participants presented with delays, however, it cannot be overlooked that some domain- (communication: 13.6%; daily living skills: 7.4%; socialisation: 18.5%; and motor: 6.2%) and subdomain-specific delays (receptive language: 17.3%; gross motor: 7.4%) were identified in this sample. Early delays may increase with age^8^ and possibly impact later academic and vocational success^9^. Delays should be addressed early in life to optimise neural plasticity, so that the negative effects of risks and delays can be ameliorated^31^. Early developmental screening can be employed to facilitate early detection and intervention for developmental delays in vulnerable children^9^. Preventative and timeous intervention enhances early developmentally-sensitive periods and minimises long-term impairments, providing infants, families, and communities with developmental, educational, and economic benefits^9^,^14^. It is therefore recommended that health professionals prioritise developmental monitoring and early identification of delays in vulnerable infants from low-income contexts, irrespective of HIV and ARV exposure. Early intervention initiatives for vulnerable populations may include caregiver information sessions while infants are waiting in line for well-baby visits. Furthermore, staff training opportunities at primary healthcare clinics provide an avenue for early interventionists to highlight the importance of ongoing developmental surveillance.

The findings, although based on a small sample, are valuable for early intervention clinicians. The two groups were comparable in terms of demographic characteristics, strengthening the findings. Another strength was the use of a valid and reliable assessment tool which is commonly used for diagnosing developmental delays^17^. Large-scale longitudinal studies monitoring infant development until a HIV-status is confirmed, will be valuable.

## Conclusion

The current study contributes to the pool of research suggesting that the developmental outcomes of HE infants during late infancy do not differ significantly from their HU peers. Developmental differences may only emerge with increasing age^8^, and therefore continued longitudinal research efforts on the developmental needs of the HE population is recommended. In addition, delays were present in both groups, suggesting that the entire sample was a vulnerable group requiring developmental monitoring. The study highlights the importance of developmental surveillance and early intervention for all infants in low-income contexts, irrespective of their HIV and ARV exposure status.
